# Mississippi River and Sea Surface Height Effects on Oil Slick Migration

**DOI:** 10.1371/journal.pone.0036037

**Published:** 2012-04-27

**Authors:** Frederico Falcini, Douglas J. Jerolmack, Bruno Buongiorno Nardelli

**Affiliations:** 1 St. Anthony Falls Laboratory, National Center for Earth-surface Dynamics, University of Minnesota, Minneapolis, Minnesota, United States of America; 2 Department of Earth and Environmental Science, University of Pennsylvania, Philadelphia, Pennsylvania, United States of America; 3 Istituto di Scienze dell' Atmosfera e del Clima, Consiglio Nazionale delle Ricerche, Rome, Italy; 4 Istituto per l'Ambiente Marino Costiero, Consiglio Nazionale delle Ricerche, Naples, Italy; University of Vigo, Spain

## Abstract

Millions of barrels of oil escaped into the Gulf of Mexico (GoM) after the 20 April, 2010 explosion of Deepwater Horizon (DH). Ocean circulation models were used to forecast oil slick migration in the GoM, however such models do not explicitly treat the effects of secondary eddy-slopes or Mississippi River (MR) hydrodynamics. Here we report oil front migration that appears to be driven by sea surface level (SSL) slopes, and identify a previously unreported effect of the MR plume: under conditions of relatively high river discharge and weak winds, a freshwater mound can form around the MR Delta. We performed temporal oil slick position and altimeter analysis, employing both interpolated altimetry data and along-track measurements for coastal applications. The observed freshwater mound appears to have pushed the DH oil slick seaward from the Delta coastline. We provide a physical mechanism for this novel effect of the MR, using a two-layer pressure-driven flow model. Results show how SSL variations can drive a cross-slope migration of surface oil slicks that may reach velocities of order km/day, and confirm a lag time of order 5–10 days between mound formation and slick migration, as observed form the satellite analysis. Incorporating these effects into more complex ocean models will improve forecasts of slick migration for future spills. More generally, large SSL variations at the MR mouth may also affect the dispersal of freshwater, nutrients and sediment associated with the MR plume.

## Introduction

**Figure 1:**
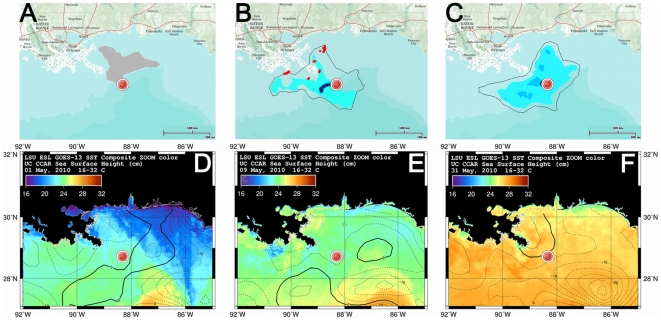
Surface slick position, SSH, and SST patterns. Top: approximate location of the oil slick provided by ESRI (http://www.esri.com/services/disaster-response), for the days (A) 1 May, (B) 9 May, and (C) 31 May 2010, respectively. Blue colors correspond qualitatively to slick intensity, while red×symbols show locations of oil slick landfall. Bottom: Overlay maps of GoM SSH (from Jason, TOPEX/Poseidon (T/P), Geosat Follow-On (GFO), ERS-2 and Envisat altimeter real-time data - Colorado Center for Astrodynamics Research) and NOAA/AVHRR SST data (Earth Scan Lab - Coastal Studies Institute, Louisiana State University). SSH contour interval is 5 cm; thick contour indicates 0 cm; (D), (E) and (F) correspond to (A), (B) and (C), respectively. Red dot indicates DH site.

Tracking the dispersal and break down of all components of oil following a spill is important for assessing the damage and recovery of ecosystems and fisheries [1]. The surface oil slick, however, is the most visible part of an oil spill, and satellite observations provide a wealth of data relevant to its migration. As an example, sea surface temperature (SST), altimeter sea level anomalies (SLA, estimated as sea surface height anomalies with respect to a temporal mean), and surface oil slick position, are displayed in Figure 1, and Figure S1. Regional ocean circulation models, which model the barotropic and baroclinic motions of sea water in the GoM, were called upon to forecast the migration of the DH oil slick (Text S1). Although researchers are modifying these models to improve such a prediction, many of them do not currently incorporate some physical properties of the slick, such as its buoyancy effects, that may be important for migration. In addition, ocean circulation models typically employ a simplified treatment of river outflows that might not capture baroclinic or backwater effects occurring off the river mouth [2], [3]. Researchers are now developing more sophisticated treatments of river plume dynamics [4], [5], however these models have not yet been deployed for oil slick dynamics purposes in the GoM. The MR plume can indeed exert a strong influence on circulation and sedimentation patterns in the northern GoM [6], [7], [8], [9], [10], [11]. Spreading and seaward penetration of the plume is dominated by the magnitude of river discharge, followed by wind stress and the effects of eddy currents [7], [8], [12]. Because the freshwater river plume is buoyant, its dynamics can be affected by local SSL patterns [8], [9], [10]. The MR river plume, which is characterized by a low spreading rate [2], [12], in turn can itself generate a vertical freshwater mound, i.e., a sea surface height anomaly around the river mouth due to buoyancy, momentum, and baroclinic effects [3], [4], [13], [14], [15], which is recognizable in both altimeter derived SLA and absolute dynamic topography (ADT) data (obtained by referencing measured sea surface height with respect to a synthetic estimate of the geoid).

*Walker et al.* [8] suggested that, due to its buoyancy, an oil slick may be strongly affected by variations in sea surface elevation: frontal zones recognized in SLA and SST patterns [16], [17] may thus constitute efficient traps or natural booms for spilled oil. This work stresses the need to better understand the additional contribution that SSL patterns exert on the movement of oil spills, and in particular whether the MR plume may affect mesoscale SSL patterns.

## Results

### Preliminary observations

In the early weeks of the DH spill (the end of April/beginning of May, 2010) we identified – by preliminarily analysis of CCAR Sea Surface Anomaly interpolated data – an East-West trending “ridge" of positive SLA, possibly related to mesoscale slope eddies [8], [16], [17], which appeared to be arresting southward spread of the oil slick (Figure 1A, D). Around May 8–9, a “valley" opened in the ridge just south of the DH site and the slick rapidly expanded southward (Figure 1B, E). This “ridge effect" demonstrates an influence of SSL (and thus of SST fronts [16], [17]) that was not captured by ocean circulation models – stressing the role of non-geostrophic, cross-slope effects [8], [18] on oil slick dynamics.

**Figure 2:**
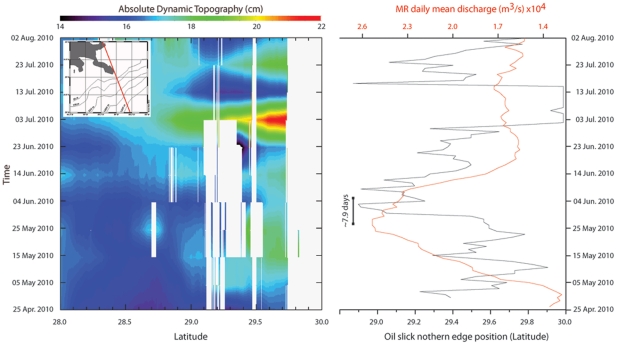
Hovmöller plot of along-track ADT measurements (see SI) matched with time series of Mississippi discharge (m^3^/s) at Belle Chasse, LA (data from USGS – National Water information System), and the northern edge position of the oil slick (data from ESRI as in **Figure 1**), 25 April to 2 August, 2010. The upper-left box indicates the portion of JASON2 track 204 considered in this work.

It has been suggested that high discharge from the MR during Spring 2010 helped to keep the surface slick away from the coast of Louisiana [19], although no mechanism was cited. Some proposed to manage flows between the two MR Delta channels in order to maximize this effect to protect fragile wetlands [20]. Discharge from the MR gradually increased over the month of May 2010, and crested for several days from late May to early June (Figure 2). The surface slick was making landfall in coastal Louisiana for several weeks, however beginning 25 May 2010 it detached from the shoreline of the Birdsfoot Delta and migrated SE out to sea at a speed of ∼2×10^−2^ m/s (Figure 1C, Figure 2). The slick remained detached from the shoreline for more than one week (Figure 2). Satellite data from this time show a SST front associated with an SLA slope surrounding the Birdsfoot Delta (Figure 1F, Figure S1, Figure 2).

### Time series and along-track coastal altimeter analysis

SLA patterns observed in the near-shore environment from the previous analysis (Figure 1) must be interpreted with caution: the interpolated altimetry data considered have large uncertainty and do not include the contribution of the mean currents on the surface elevation [21]. For this reason we performed a more rigorous analysis of sea surface topography, and corresponding oil slick dynamics, using time-series data over the period of 25 April to 2 August, 2010. The analysis of coastal sea-surface altimetry was based on along-track ADT measurements obtained by the PISTACH (Prototype Innovant de Système de Traitement pour les Applications Côtières et l'Hydrologie) project [22] using Jason2 satellite track 204 (Text S1).

**Figure 3:**
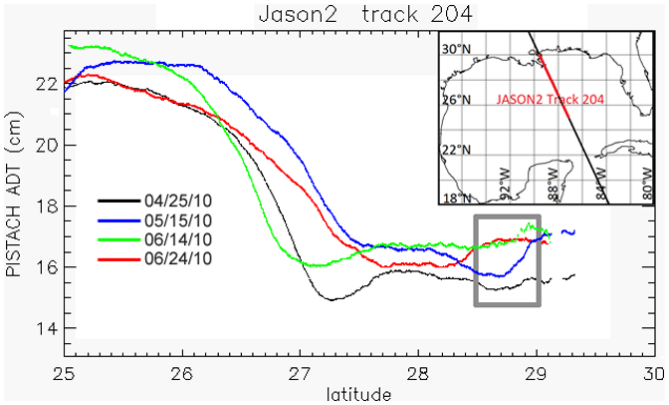
Filtered along-track ADT measurements for the days 25 April, 15 May, 14 June and 24 June, 2010. The upper-right box indicates the portion of JASON2 track 204 considered in this plots. The gray box indicates the region off the Mississippi Birdsfoot, where the freshwater mound formation occurred. Notice how the ADT rises from 25 April to 14 June for the track portion 27.5°N–29.5°N; then the ADT begins to fall. The highest slope occurs at 15 May, i.e., around the peak of the MR discharge as shown in Figure 2 (see Table 1).

Tracking oil slick migration is also difficult because available surface maps for the DH spill are somewhat subjective [18]. We tracked the latitudinal position of the northern edge of the oil slick, identified from published maps of daily slick extent (Figure 1). We also performed analysis for the southern edge and centroid of the slick, which produced similar results. Taken together, data suggest the following scenario (Figure 2). High MR discharge from approximately 10 May to 10 June caused an increase of the ADT in the vicinity of the Birdsfoot, generating ∼4×10^−2^ m of relief over a distance of ∼70×10^3^ km, resulting in a seaward-directed sea-surface slope of approximately ∼10^−6^ (Table 1; Figure 3). The scale of this freshwater mound is consistent with expectations for a subcritical, non-diffusive plume [12], based on numerical and experimental results [23], [24]. Creation of this incline resulted in a cross-slope, seaward migration of the northern boundary of the oil slick at a speed of ∼2×10^−2^ m/s (Figure 2). This physical mechanisms will be tested and discussed later on. Cross-correlation analysis shows that oil slick migration lagged MR discharge by ∼8 days (Figure 2), with a correlation coefficient of *r*∼0.363. By using the critical value table for Pearson's Correlation Coefficient, we found that *r*>*r_m_*, where *r_m_* = 0.273 is the minimum correlation coefficient that one needs to confidently state that the relationship is statistically significant. The arrival of Hurricane Alex (25 June–2 July, 2010) generated strong onshore winds, causing a large increase in ADT along the Louisiana coast during a period of low MR discharge (Figure 2). Large wind stresses resulting from the hurricane appear to have pushed the oil slick landward (Figure 2, Figure S3).

**Table 1:** SLA and ADT gradients evaluated from CCAR (Figure 1) and PISTACH data (Figure 2, Figure 3), respectively, along the track 204 of Jason 2.

	CCAR (SLA)	PISTACH (ADT)
25-Apr-2010	5.0×10^−7^	8.0×10^−8^
14-May-2010	1.5×10^−6^	2.5×10^−7^
14-Jun-2010	2.5×10^−6^	1.5×10^−7^
24-Jun-2010	5.0×10^−7^	5.0×10^−8^

Gradients have been evaluated by considering the difference in elevation between 29.5°N and 27.5°N and then dividing by the distance along the track. Such a region off the Mississippi River Bridsfoot represents the area where the freshwater mound formation occurs.

**Figure 4:**
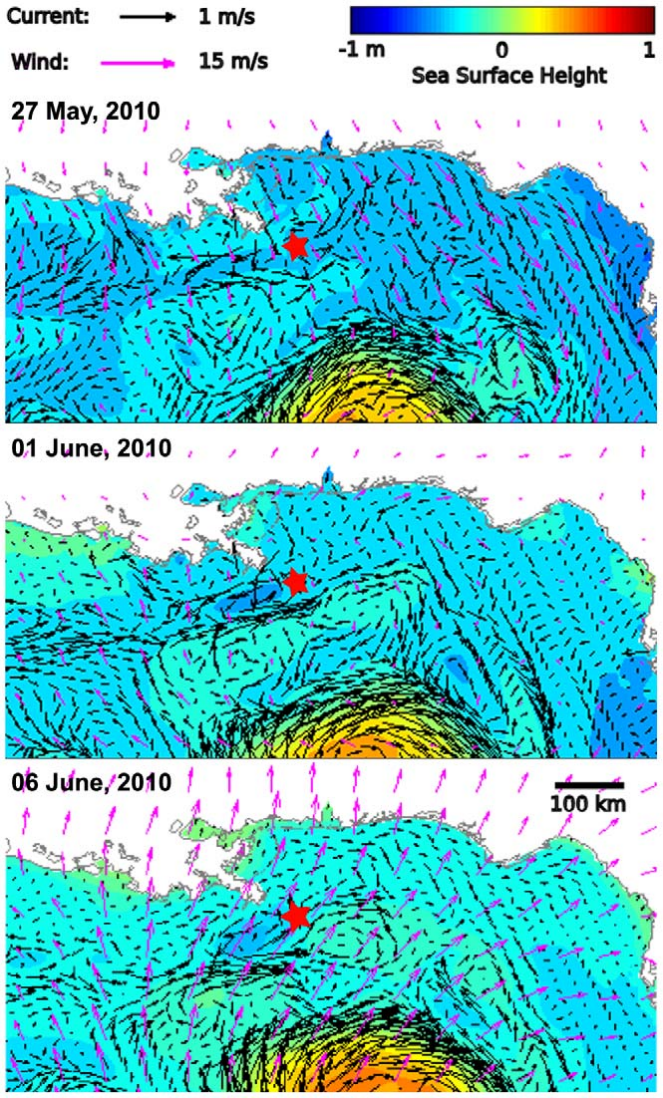
Nowcast results from South Atlantic Bight and GoM Circulation (SABGOM) model showing simulated ocean currents (black vectors) and SSH (color) for indicated dates. Superimposed are wind vectors (pink) at 10 m above ground level, generated from 12 km re-analysis data, NOAA Air Resources Laboratory NAM12. Note that neither winds nor simulated currents (Figure S2) correspond to observed slick movement for the MR high discharge event, and also the disagreement between measured and modeled SSH (Figure 1, Figure 2).

Although near-shore water circulation in the northern GoM is mainly driven by winds [8], the seaward slick migration observed from 25 May to 10 June (Figure 2) cannot be explained by measured surface winds, which were weak and moving onshore until the passage of Hurricane Alex (Figure 4). Migration was also counter to the along-shore motion that would be expected by a quasi-geostrophic current [25] and that was forecast by modeled ocean currents (Figure 4, Figure S2). Although model forecasts include temperature and salinity inputs from the MR, they failed to capture the secondary effect of a MR front (Figure 4, Figure S2) because they do not consider additional cross-shelf river effects that may substantially influence the local SSL. Both the interpolated altimetry data (Figure 1) and the along-track measurements for coastal applications (Figure 2, Figure 3) show that, under some conditions, the MR generates local gradients in sea surface elevation that are many times larger than those predicted from several numerical models (Figure S2, Text S1). River-dedicated models can capture the correct physics to resolve variations in SSL due to a river outflow [5]. However, they were not used for forecasting oil slick dynamics in the GoM.

Creation of the MR freshwater mound is likely due to a combination of channel geometry, hydrologic, momentum, and baroclinic effects [4], [12], [14], [21], [23], [24], [25], in addition to wind and offshore ocean conditions. However, it can be understood broadly as the seaward extension of the water-surface slope of the MR itself: during flood stage, the lower MR has a characteristic slope of *S* = 10^−6^–10^−5^ [2], [26], [27], consistent with the ADT gradient observed from 25 May, 2010. For a characteristic flood, stage at the MR mouth increases on the order of *H* = 1 m (Text S1); thus, the expected seaward extent of the plume would be of the order *H*/*S*∼100×10^3^ m, in broad agreement with the observed SLA/ADT patterns (Figure 1F, Figure 2, and Figure S4C) and hydraulic modeling [27]. Such an offshore protrusion of the river slope is further justified by the very low spreading that characterizes the MR plume [12].

While it is true for the 25 May–10 June event that the MR freshwater mound occurred during a period of high discharge, high discharge alone does not appear to be sufficient for generating a persistent freshwater mound in SSL at the River mouth [5]. The mound formed during conditions of mild winds and fairly weak near-shore currents (Figure 4). It may be that MR SSL effects are strong under quiescent shelf conditions, when MR water is not rapidly dispersed by wind and eddy currents. The Hurricane Alex example demonstrates that strong wind stresses can overwhelm other effects and dominate the movement of the surface oil slick, destroying any correlation between the MR and oil slick dynamics.

### The multi-layered film flow model

It has recently been recognized that a complex pattern of barriers and conduits in the GoM may drive oil slick migration in a manner that departs from the sea surface ocean circulation [18]. The analysis here is strongly suggestive that MR SSL effects may be an important component. However, the time series is of limited duration and, furthermore, does not provide a physical mechanism. Thus, interpretations derived from satellite observations alone (Figures 1, 2, and 3) are suggestive but equivocal. Here we propose that SSL contributes a pressure-driven flow component to surface slick cross-slope movement, and use a multi-layered film flow model [28], [29] to test this hypothesis and validate the above analysis. A freshwater mound such as that seen from 25 May to 10 June, 2010 would generate strong pressure gradients [23], [24]. Although localized, such gradients could have had a large impact on slick dynamics because of the proximity of the DH spill to the MR mouth (Figure 1, Figure 2). Given the environmental complexity in the GoM, and the uncertainty of both ocean model forecasts and the actual extent of the DH surface slick, it is difficult to quantitatively assess the effect of the MR freshwater mound on slick migration. Therefore our model tests the expected magnitude of this SSL effect on a buoyant slick (Text S1), for conditions representative of the MR high discharge event.

**Figure 5:**
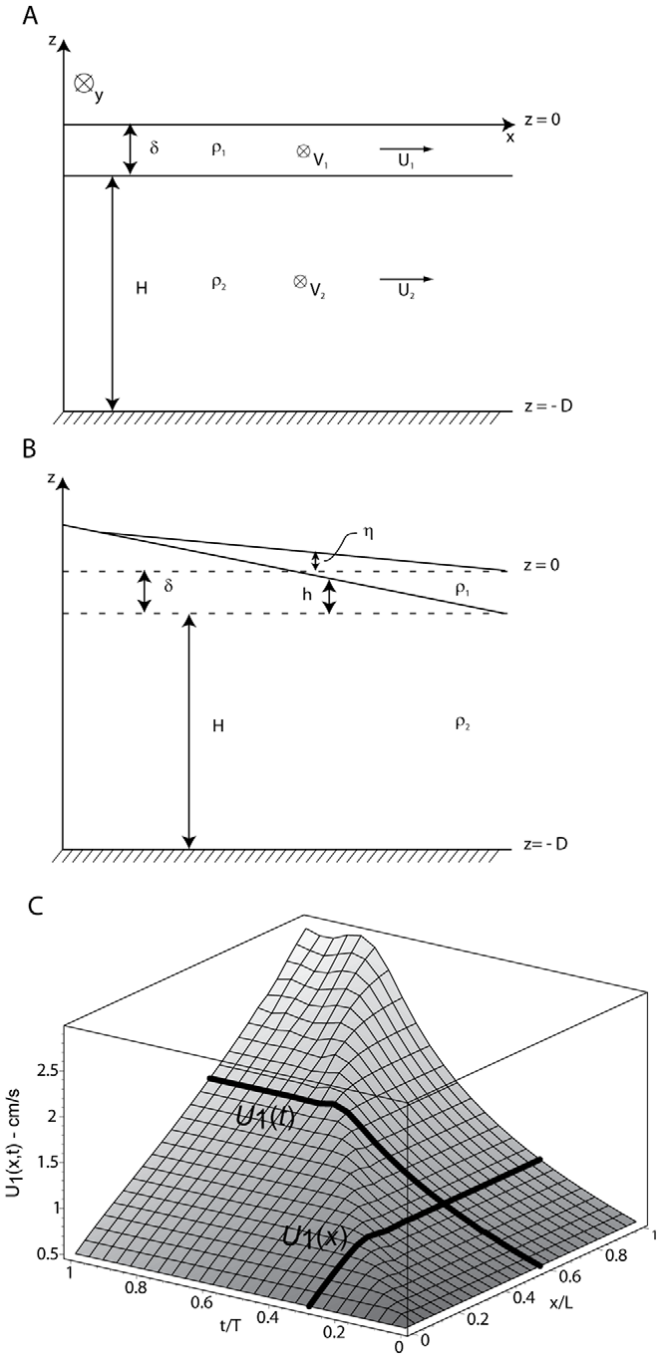
Schematic representation of the two-layer model. It shows (A) all symbols used in the text: thin oil layer of density ρ_1_, thickness δ, seaward velocity *U*_1_, and alongshore velocity *V*_1_, laying above a water layer characterized by ρ_2_, *H*, *U*_2_ and *V*_2_. *x* and *y* coordinates are directed seaward and along-shelf, respectively. *D* is depth of the continental shelf. (B) Layer pattern under a mound effect, where now thickness is δ+η and *H*+*h* for the upper and bottom layer, respectively. (C) Solution for equation (1) in space (*x*) and time (*t*). Two bold lines show representative oil slick seaward velocity at a fixed location evolving through time (*U*(*t*)), and a spatial seaward transect at a fixed time (*U*(*x*)). For example, at the given location *x/L* = 0.5, *U* increases with time until it achieves a steady state at *t/T* = 0.4. Coordinates are normalized by *L* = 10^4^ m and *T* = 10^6^ s (see Text S1).

Considering a viscous two layer system in a rotating frame [28], [29], [30], [31], in which a thin upper layer of less-dense oil sits above a static homogeneous layer of ocean shelf water (Figure 5), we derive the coupled momentum equation: in a shallow water approximation the adjusted pressure for both layers is therefore given by [29], [30], [31]

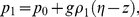
(1a)

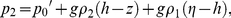
(1b)where *p*_0_ and *p*_0_' are constant, *g* is gravity, *h* = *h*(*x*) and *η* = *η* (x) are the thickness anomalies of the water and oil layer, respectively, due to the MR tilting effect (Figure 5).

From equations (1), the shallow water cross-shelf momentum equations in the unsteady and viscid case for each layer are [28], [29], [30], [31]:
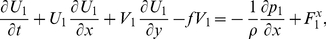
(2a)

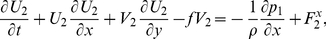
(2b)where *U_i_* and *V_i_* (*i* = 1,2) are the cross- and along-shelf velocity components of both layers, respectively; *f* is the Coriolis parameter and *F_i_^x^* represents the external forces acting on both layers along cross-shelf component *x* (Figure 5). Note that, according to the Boussinesq approximation, *ρ* in equations (2) is a mean density (Text S1). For sake of simplicity, and since they will not be used in further analysis, we do not report here along-shore (*y*-direction) momentum equations. It should be noted, however, that 2D flow effects could easily be incorporated into this framework.

By substituting *p*_1_ and *p*_2_ of equations (1) in equations (2), and subtracting the momentum equation of the upper layer from that of the lower layer (see a complete description of the algebra in Text S1) one obtains
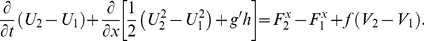
(3)Equation (3) describes the oil slick velocity (*U_1_*) along the seaward direction (*x*) that results from SSL spatial variations, here considered as 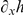. Here 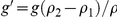 is the reduced gravity, *ρ* the mean density, *g* the gravity. 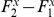 can be expressed as ∼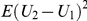, where *E* is a drag coefficient that dynamically couples the oil slick with the water surface (Text S1).

We seek to describe the role of river tilting of the SSL in detaching the oil slick from the shore, which corresponds to a positive offshore velocity *U*_1_. Therefore in equation (3) we assume *U*_2_ = 0 and *V*_2_ = 0. Superimposing a function *h* = *h*(*x*) that roughly approximates the water surface tilting due to the MR freshwater mound (Table 1, Figure 3), equation (3) gives the spatial and temporal evolution of the seaward oil slick velocity (Figure 5) that would result from the pressure field caused by the SSL anomaly. The proposed model shows a realistic solution related to the freshwater mound effect: the oil slick increases its seaward (i.e., cross-slope) velocity both in space and time, eventually achieving a steady offshore migration after a lag time that depends on the drag coefficient (Text S1). For a drag coefficient ∼10^−3^ m^−1^, which was estimated from a momentum balance analysis and by assuming that the surface layer moves at approximately ∼10^−2^ m/s (Figure 2, Text S1), the model predicts a lag time of order 5–10 days (i.e., ∼10^5^ s) between mound formation and slick shoreline detachment (Figure 5C). This is in good agreement with measured slick dynamics of the MR high discharge event (Figure 2). Thus the model appears to be relevant for predicting oil slick migration due to MR hydraulic control once a realistic drag coefficient can be set (Text S1). Pressure-driven flow magnitudes are comparable to modeled ocean current velocities, but differ in their direction. It is therefore possible that some component of oil slick movement is independent of the underlying surface ocean currents, in the presence of SSL/SST fronts [8], [16], [17]. This decoupling may be enhanced by the suppression of wind-driven transport, due to reduction of sea surface roughness by oil slicks [32]. Our analysis indicates that SSL and wind patterns competed to drive migration of the DH oil slick (Text S1).

## Discussion

Long-term forecasting of oil slick dispersion requires consideration of many physical, chemical and biological processes [28], [32], [33]. For long and continuous oil spills such as DH, however, daily predictions of slick migration are also needed to divert limited resources to coastal areas that need them most. Spills such as DH may happen again as oil exploration continues to push into deeper waters [34]. Our results show that some of the migration patterns of the DH surface slick were driven by previously unrecognized sea surface slope effects, confirming observations of secondary slick propagation toward mixing zones [18] that differs from the main sea surface circulation. Gradients in SSL can both drive and retard the movement of surface oil slicks (Figure 1, Figure 2). Although we focused on novel MR effects, wind and ocean circulation also can drive pressure-driven movement of surface slicks (Figure 1B,E, Figure S4) that may be modeled using the approach presented here. Ocean models in the near future will include a more realistic representation of the fresh-water mound [4], [5]. Computing pressure-driven flow effects from these simulations seems like a particularly fruitful path forward in forecasting slick migration under the combined influence of rivers and ocean currents. Finally, the MR freshwater mound may be an important but unrecognized factor in determining the dispersion of the buoyant MR plume itself and its associated nutrients and sediment.

## Materials and Methods

### Oil slick location

Approximate location of the oil slick was provided by ESRI (Figure 1) and was derived from satellite data published by NOAA National Ocean Service – Office of Response and Restoration. The application was powered by ArcGIS (http://www.esri.com/software/arcgis/) and shows the locations of the oil slick in the GoM for each day from April 25, when the slick was first measured, to August 4, when static kill was successful. It also shows oil slick projections, as well as locations of critical habitats (Figure 1).

### Sea surface temperature

SST data are recorded by the Advanced Very High Resolution Radiometer (AVHRR), a sensor operating onboard of the NOAA - POES series (Polar-Orbiting Operational Environmental Satellites). The AVHRR sensor is a radiation-detection imager that can be used for remotely determining the surface temperature of a body of water. This scanning radiometer uses 6 detectors that collect different bands of radiation wavelengths with a resolution all of 1.1 km. Along-track wavelength data have to be processed and interpolated in order to obtain SST high resolution maps. For our work we used maps provided by the Earth Scan Laboratory of the Louisiana State University (http://www.esl.lsu.edu/home/). The MR plume around the Birdsfoot (Figure 1, Figure S1) can be recognized from the temperature contrast between the sea water and the fresh, colder river water.

### River discharge

In order to investigate the potential role of the MR in the southward migration of the oil slick, we examined USGS surface-water time-series data for streamflow (i.e., discharge). These data (http://waterdata.usgs.gov/usa/nwis/sw) are collected by automatic recorders and manual measurements at field installations.

### Sea level anomalies

We first used data from the CCAR Global Near Real-Time Sea Surface Anomaly Data Viewer. Maps are produced from Jason, Geosat Follow-On(GFO), and Envisat altimeter data processed in near real-time, usually within 12 to 36 hours of overflight. An analysis product is based on the latest 10 days of satellites sampling.

### Coastal Altimeter data

The altimeter products used for our detailed coastal analysis are the along-track measurements produced by the PISTACH (Prototype Innovant de Système de Traitement pour les Applications Côtières et l'Hydrologie) project [22]. These data have been developed as an experimental evolution of the AVISO (Archiving, Validation and Interpretation of Satellite Oceanographic data) Jason-2 Interim Geophysical Data Record (IGDR) products [35]. They are produced by applying algorithms specifically designed for coastal applications, and include state-of-the-art geophysical corrections (Text S1).

## Supporting Information

Figure S1 **NOAA/AVHRR SST data related to Mississippi River high-discharge event.** (A) 1 June and (B) 6 June, 2010 (data processed by the Earth Scan Lab - Coastal Studies Institute, Louisiana State University). Panels have different ranges in scale. Note cool colors corresponding to fresh MR water surrounding the Birdsfoot.
(TIF)

Figure S2 **NRM IASNFS model nowcast** [**3**] **for 0000 UTC 27 May, 1 June, and 6 June 2010 (A, B, and C, respectively) of SSL and (D, E, and F, respectively) SSS.** IASNFS consists an 1/24 degree, 41-level sigma-z data-assimilating ocean model based on NCOM. The model assimilates the synthetic temperature/salinity profiles generated by a data analysis model called MODAS to produce nowcast. Real-time data come from satellite altimeter (Jason-1, ERS-2) SLA and AVHRR SST. Three hourly surface heat fluxes, including solar radiation, wind stresses and sea level air pressure from NOGAPS/FNMOC are applied for surface forcing. The open boundary conditions including SSL, transport, temperature, salinity and currents are provided by the NRL 1/8 degree Global NCOM which is operated daily [20]. Note in panels A, B, C the disagreement between modeled SLA and measured (Figure 2) ADT in the vicinity of the Birdsfoot (the outermost part of the Mississippi Delta, NW of the Deepwater Horizon location).
(TIF)

Figure S3 **Oil slick extent (left) and ADT data (right) for the time period following Hurricane Alex's passage, 2–11 July 2010.** Approximate location of the oil slick provided by ESRI (http://www.esri.com/services/disaster-response), derived from satellite data published by NOAA National Ocean Service – Office of Response and Restoration. Blue colors correspond qualitatively to slick intensity, while red×symbols show locations of oil slick landfall on a given day. Surface slick location and concentration are difficult to quantify, so these maps are meant for illustrative purposes only. NAM12 winds are also shown in inset of right column. Note stronger onshore winds push oil slick “uphill" against the SSH gradient in early July, but as winds subside the slick recedes out to sea along SSH gradients.
(TIF)

Figure S4 **Cross-shelf vertical section of sea temperature from the WFSROMS model.** (A) for a low MR discharge period (1 May 2010) and (B) a high MR discharge event (6 June 2010). Geographical position of the vertical section is shown in the boxes. (C) Schematic representation of the MR water surface slope (solid line), and its elevation (H) and seaward extension (L) during floods (dashed line).
(TIF)

Text S1 **Ocean model results, coastal altimeter data, and the two-layer flow model.**
(DOC)
