# Supplementary figures and images for: Mississippi River and Sea Surface Height Effects on Oil Slick Migration
Source: PLoS One. 2012 Apr 27;7(4):e36037. doi: 10.1371/journal.pone.0036037 (PMC3338853; doi:10.1371/journal.pone.0036037)

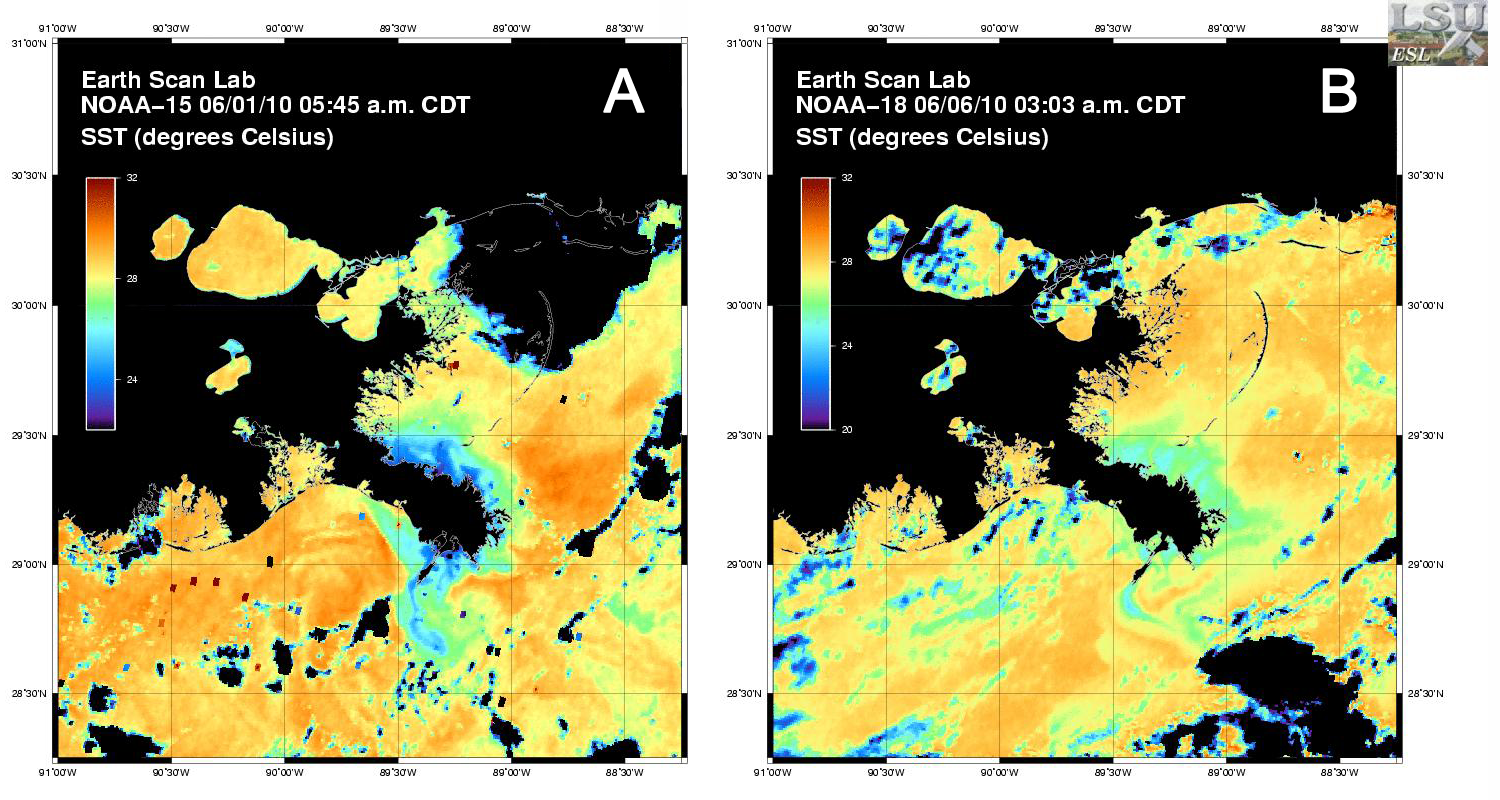

Supplement: Figure S1 — NOAA/AVHRR SST data related to Mississippi River high-discharge event. (A) 1 June and (B) 6 June, 2010 (data processed by the Earth Scan Lab - Coastal Studies Institute, Louisiana State University). Panels have different ranges in scale. Note cool colors corresponding to fresh MR water surrounding the Birdsfoot. (TIF) [file pone.0036037.s001.tif]

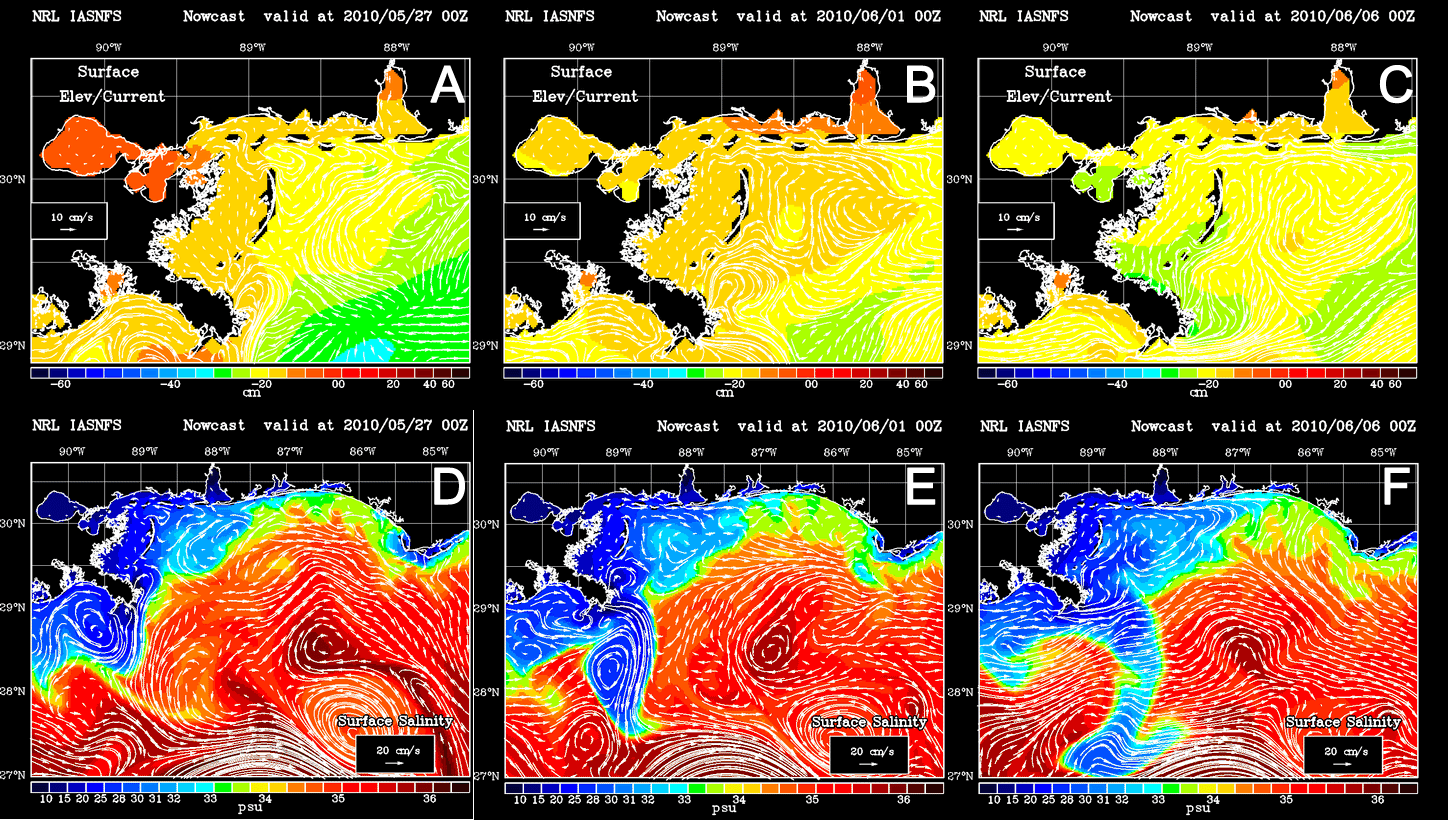

Supplement: Figure S2 — NRM IASNFS model nowcast [3] for 0000 UTC 27 May, 1 June, and 6 June 2010 (A, B, and C, respectively) of SSL and (D, E, and F, respectively) SSS. IASNFS consists an 1/24 degree, 41-level sigma-z data-assimilating ocean model based on NCOM. The model assimilates the synthetic temperature/salinity profiles generated by a data analysis model called MODAS to produce nowcast. Real-time data come from satellite altimeter (Jason-1, ERS-2) SLA and AVHRR SST. Three hourly surface heat fluxes, including solar radiation, wind stresses and sea level air pressure from NOGAPS/FNMOC are applied for surface forcing. The open boundary conditions including SSL, transport, temperature, salinity and currents are provided by the NRL 1/8 degree Global NCOM which is operated daily [20]. Note in panels A, B, C the disagreement between modeled SLA and measured (Figure 2) ADT in the vicinity of the Birdsfoot (the outermost part of the Mississippi Delta, NW of the Deepwater Horizon location). (TIF) [file pone.0036037.s002.tif]

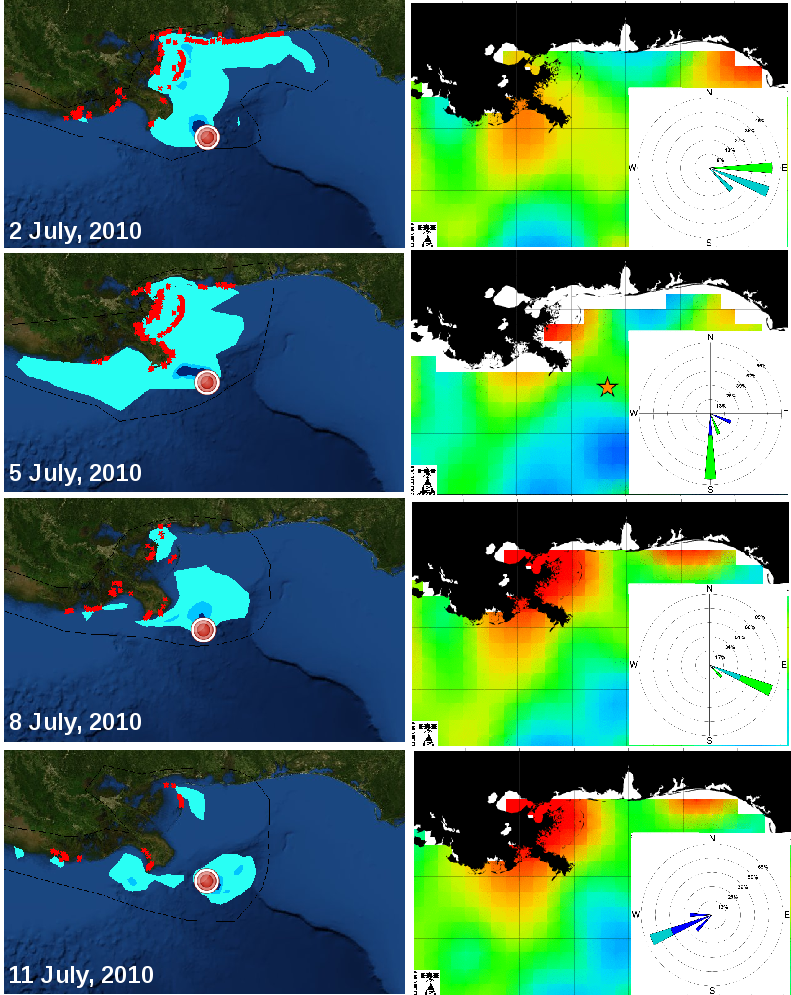

Supplement: Figure S3 — Oil slick extent (left) and ADT data (right) for the time period following Hurricane Alex's passage, 2–11 July 2010. Approximate location of the oil slick provided by ESRI (http://www.esri.com/services/disaster-response), derived from satellite data published by NOAA National Ocean Service – Office of Response and Restoration. Blue colors correspond qualitatively to slick intensity, while red×symbols show locations of oil slick landfall on a given day. Surface slick location and concentration are difficult to quantify, so these maps are meant for illustrative purposes only. NAM12 winds are also shown in inset of right column. Note stronger onshore winds push oil slick “uphill" against the SSH gradient in early July, but as winds subside the slick recedes out to sea along SSH gradients. (TIF) [file pone.0036037.s003.tif]

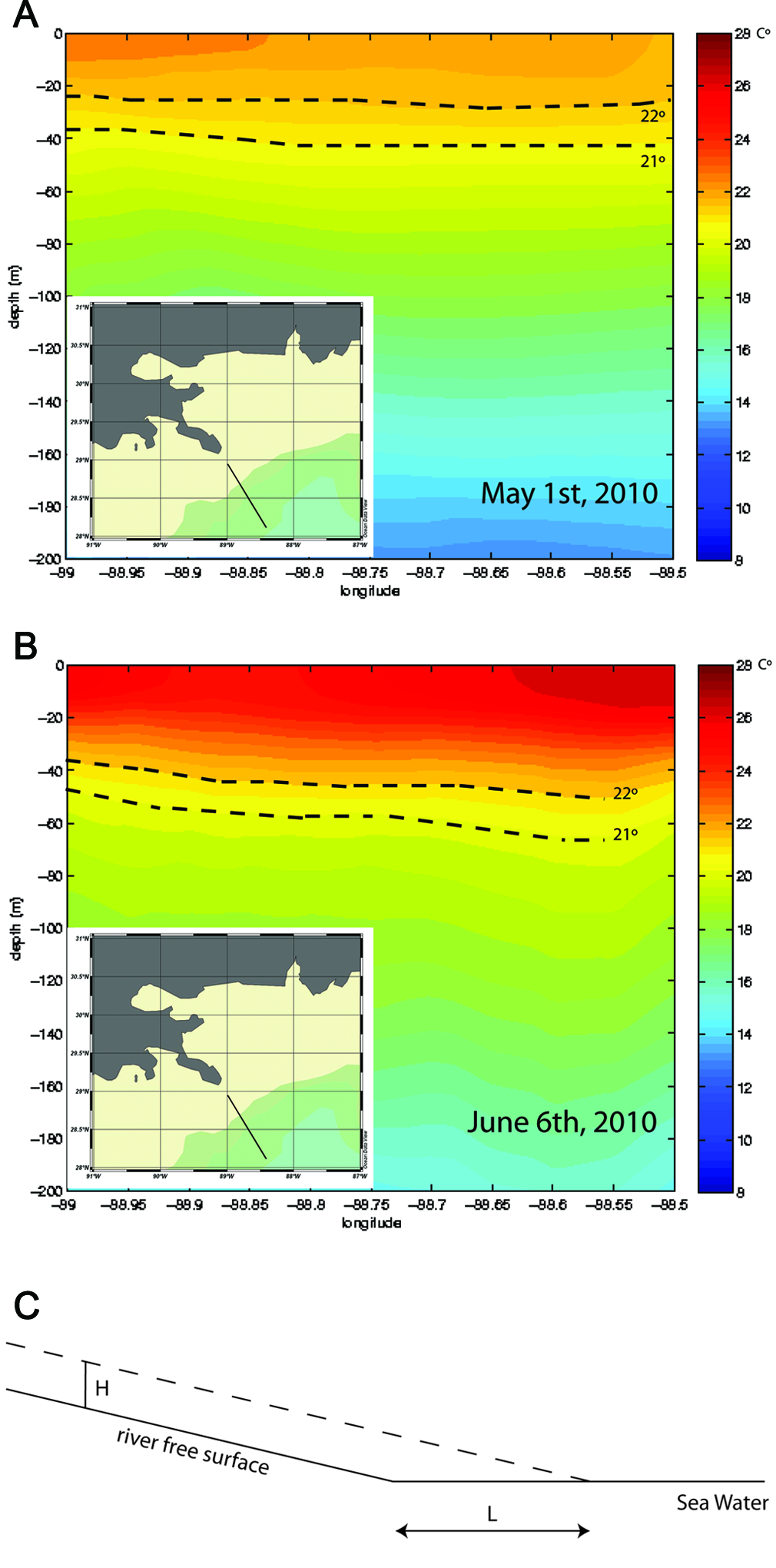

Supplement: Figure S4 — Cross-shelf vertical section of sea temperature from the WFSROMS model. (A) for a low MR discharge period (1 May 2010) and (B) a high MR discharge event (6 June 2010). Geographical position of the vertical section is shown in the boxes. (C) Schematic representation of the MR water surface slope (solid line), and its elevation (H) and seaward extension (L) during floods (dashed line). (TIF) [file pone.0036037.s004.tif]
