# Supplementary material for: Mississippi River and Sea Surface Height Effects on Oil Slick Migration
Source: PLoS One. 2012 Apr 27;7(4):e36037. doi: 10.1371/journal.pone.0036037 (PMC3338853; doi:10.1371/journal.pone.0036037)
Supplement: Text S1 — Ocean model results, coastal altimeter data, and the two-layer flow model. (DOC) [file pone.0036037.s005.doc]

**SUPPLEMENTARY INORMATION**

**Mississippi River and sea surface height drive migration of surface oil slick**

*by F. Falcini and D.J. Jerolmack and B. Buongiorno Nardelli*

**Supporting additional data and ocean model results**

High discharge from the Mississippi River (MR) during early June 2010 coincided with a positive sea surface level anomaly (SLA) at the River mouth, and a related sea surface temperature (SST) front (Figure 1C, Figure 1F, Figure S1). This event appeared to affect the surface oil slick of the Deepwater Horizon (DH) spill, pushing the slick seaward such that it detached from the shoreline of the Birdsfoot (Figure 2). We compare nowcasts/forecasts of ocean currents, sea surface salinity (SSS) and SSL for the MR high discharge event by considering data of 27 May, 1 June, and 6 June, 2010 from the following models: South Atlantic Bight and Gulf of Mexico Circulation [1] (SABGOM, Figure 3), West Florida Shelf Regional Ocean Modeling System [2] (WFSROMS, not shown), and Naval Research Laboratory Intra-Americas Sea Nowcast/Forecast System [3] (NRL IASNFS, Figure S2).

Although there are differences in the details of different model forecasts, the general patterns of currents, SSS and SSL are comparable. Such regional ocean circulation models, which model the barotropic and baroclinic motions of sea water in the Gulf of Mexico (GoM), predict flows that are generally in a quasi-geostrophic balance. The freshwater mound of the MR plume, represented by both the altimeter SLA and ADT data around the Birdsfoot (Figure 1, Figure 2), was not captured by any of the models. All three models predicted a topographic trough in the vicinity of the MR mouth, rather than a mound (Figure 4, Figure S2).

Measured SST (Figure S1) and modeled SSS (Figure S2) support the contention that the mound was composed of MR freshwater. Because oil is buoyant, the pressure gradients created by the MR SSL anomaly would cause a non-geostrophic seaward (cross-slope) migration of the surface slick. Indeed, seaward movement and detachment of the surface slick from the shoreline occurred from 25 May to 10 June, 2010 (Figure 2). We develop and apply a model for this phenomenon below.

**Coastal Altimeter data**

Caution must be exercised in interpreting altimeter data on the GoM shelf close to the shoreline. The altimeter data used for this analysis are obtained from the along-track measurements produced by the PISTACH (Prototype Innovant de Système de Traitement pour les Applications Côtières et l’Hydrologie) project [4]. These data have been developed as an experimental evolution of the AVISO (Archiving, Validation and Interpretation of Satellite Oceanographic data) Jason-2 Interim Geophysical Data Record (IGDR) products [5]. They are produced by applying algorithms specifically designed for coastal applications, and include state-of-the-art geophysical corrections. PISTACH along-track data (Figure 2, Figure 3) also have a higher spatial resolution with respect to standard AVISO products (sampling at 20Hz instead of 1Hz), that allows the users to define ad-hoc spatial filtering, better suited for regional/coastal studies (20Hz data corresponds to an approximate resolution of 300 m).

***Coastal waveform retracking:*** Satellite microwave altimeters estimate the distance between the radar antenna and the sea surface (*range*) by measuring the mean return time of high frequency pulses (over 1700 pulses are emitted per second), scaled by the speed of light. The sea level estimation is then obtained through the successive application of a number of corrections, mainly needed to account for the light speed decrease within the atmosphere, and a precise knowledge of the satellite orbit (see [6] and references therein, for a full description of basic radar altimetry principles).

To get accurate estimates (i.e. keeping the range error within a few cms), standard altimeter products base their single pulse return time estimates on the echo waveforms typical of open ocean surface. This part of the processing is commonly known as waveform retracking, while range accuracy is improved by averaging the pulse return times. However, in correspondence to the along-track land/sea or sea/land transition zones, the footprint of a pulse-limited altimeter (generally between 2 km and 7 km wide) can be partly over ocean and partly over land. The power and waveform of the signal received back by the radar antenna are thus modified by the relative proportion of sea and land illuminated surfaces and by the geometry of the coast, making the use of standard products not suited for coastal studies. Basically, the waveform depends on a weighted average of the land/sea scattering coefficients, which in turn are affected by the topography relief, by the nature of the terrain, etc. (e.g. [7]). Standard products are based on 1 Hz averages, meaning their along-track spatial sampling reaches about 7 km, and first usable data are at least 7 km away from the coastline. In order to account for land-contaminated waveforms shapes, and reach the higher sampling and accuracy required by coastal applications, different projects have been funded in recent years (see [8], and references therein for a full review of theory and applications of coastal altimetry).

The retracking technique proposed by PISTACH makes an objective classification of the waveforms, and then applies specific algorithms to each class, starting from high frequency (20 Hz) measurements, leading to along-track sampling of about 300 m [4]. Waveforms classification is performed analyzing return signal shapes and identifying characteristic patterns through a neural network algorithm (up to 16 classes have been identified).

***Specific along-track processing*:** The along-track altimeter data analyzed in this paper are the sea level measurements derived from the PISTACH *range_red3_ku* estimates (specific for areas including near shore zones), applying standard corrections for the dry-troposphere error, sea state bias error, inverse barometer effect, high frequency barotropic response to atmospheric forcing, pole tide, and solid tide, and selecting specific PISTACH corrections for the ionosphere (GIM iono-correction) and wet-troposphere error (*decontaminated_wet_tropo_corr,* [9]), and the most recent geocentric ocean tide height *ocean_tide_sol3* (global model GOT 4.7). The Absolute Dynamic Topography (ADT) has then been computed by adding the Mean Dynamic Topography estimated by [10]. As the high frequency sampling along track can lead to extremely noisy observations, a final data editing/filtering procedure has been applied, following the procedure described in [11], and choosing a low-pass cut-off wavelength of 30 Km (Figure 3).

Our analysis also sheds light on the effects of the passage of Hurricane Alex (Figure 2, Figure S3), which took a storm track across southern GoM in the final days of June and the first week of July, 2010. Although there was a vertical mound along the northern Gulf Coast (created by the observed onshore winds), the winds appeared to be strong enough to push oil “upward” against this seaward SSL gradient and the slick made landfall all along the MR Delta (Figure 2). As winds subsided but the mound remained, the slick migrated radially outward from the vertical mound, in the direction of SSL gradients (Figure S3).

**The Bernoulli two-layer flow model**

We here apply a multi-layer flow model, which constitutes a synthesis of a model for steady multi-film flows down an inclined plane [12], a multi-layer hydraulic exchange model [13], and a pressure-driven multi-layer model for rotating frames [14]. We consider a general but simple two dimensional (2D), two-layer system (Figure S4) where *x* and *y* are the offshore and along shore coordinates, respectively. The upper layer, with density *ρ*_1_ and thickness δ, represents the oil slick, laying above the water surface; the shelf water is here identified as a bottom layer, considered as a unique water column of thickness *H >>* δ  and mean density *ρ*_2_ (Figure 5A).

ADT data show that the seawater surface off the MR Delta was tilted seaward during the high stage of the MR in early June 2010 (Figure 1, Figure 2, Figure 3). The corresponding pressure field in both layers would therefore be modified by isopycnal anomalies, causing a new circulation pattern that would overlap the mean coastal circulation. This pattern can be shown schematically by examining vertical temperature transects oriented cross-shelf (Figure S4): a high MR discharge increases the slope of the thermal structure offshore of the River mouth (Figure S4B). In addition, hydrologic measurements along the lower MR during flood conditions show that the water surface maintains a relatively large slope of approximately 10^-5^, and hydraulic modeling indicates that this slope extends seaward of the river mouth [15]. Thus, flood waters from the MR are expected to produce a protrusion of freshwater into the GoM that follows the gradient of the lower MR (Figure S4C). In a shallow water approximation the adjusted pressure for both layers is given by equations (1) in the main text [12,13,14]:

$p_{1}=p_{0}+g\rho_{1}\left(\eta-z\right)$, (1a)

$p_{2}=p_{0}'+g\rho_{2}\left(h-z\right)+g\rho_{1}\left(\eta-h\right)$, (1b)

where *p_0_* and *p_0_*' are constant, *g* is gravity, *h*=*h*(*x*) and *η*= *η* (x) are the thickness anomalies of the water and oil layer, respectively, due to the MR tilting effect (Figure 5B). From equations (1), the shallow water cross-shelf momentum equations in the unsteady and viscid case for each layer are:

$\frac{\partial U_{1}}{\partial t}+U_{1}\frac{\partial U_{1}}{\partial x}+V_{1}\frac{\partial U_{1}}{\partial y}-fV_{1}=-\frac{1}{\rho}\frac{\partial p_{1}}{\partial x}+F_{1}^{x}$, (2a)

$\frac{\partial U_{2}}{\partial t}+U_{2}\frac{\partial U_{2}}{\partial x}+V_{2}\frac{\partial U_{2}}{\partial y}-fV_{2}=-\frac{1}{\rho}\frac{\partial p_{1}}{\partial x}+F_{2}^{x}$, (2b)

where *f* is the Coriolis parameter and $F_{i}^{x}$ represents the external forces acting on both layers along *x*. Note that, according to the Boussinesq approximation, *ρ* in equations (2) is a mean density.

The system defined by equations (1) and (2) is based on the assumption that the oil layer is sufficiently thick such that gravity-driven slope effects overcome the drag force at the oil-water interface. Data on the thickness of the DH surface oil slick are not available, so this assumption cannot be rigorously justified; however, the validity of this approach is justified *a posteriori* by comparing modeled oil slick migration to observations.

In agreement with our goal we consider our system homogeneous along the *y* coordinate, an approximation that does not affect the generality of the main result. Thus, one has that equations (2) can be written as

$\frac{\partial U_{1}}{\partial t}+\frac{\partial}{\partial x}\left[\frac{1}{2}U_{1}^{2}+\frac{p_{1}}{\rho}\right]=F_{1}^{x}+fV_{1}$, (3a)

$\frac{\partial U_{2}}{\partial t}+\frac{\partial}{\partial x}\left[\frac{1}{2}U_{2}^{2}+\frac{p_{2}}{\rho}\right]=F_{2}^{x}+fV_{2}$, (3b)

where $B_{i}=\left[\frac{1}{2}U_{i}^{2}+\frac{p_{i}}{\rho}\right]$ are the well known Bernoulli functions, usually constant for steady and inviscid systems [13,16]. By substituting *p*_1_ and *p*_2_ in equation (3) with the aid of equation (1), our momentum equations become

$\frac{\partial U_{1}}{\partial t}+\frac{\partial}{\partial x}\left[\frac{1}{2}U_{1}^{2}+g\frac{\rho_{1}}{\rho}\eta\right]=F_{1}^{x}+fV_{1}$, (4a)

$\frac{\partial U_{2}}{\partial t}+\frac{\partial}{\partial x}\left[\frac{1}{2}U_{2}^{2}+g\frac{\rho_{2}}{\rho}h+g\frac{\rho_{1}}{\rho}(\eta-h)\right]=F_{2}^{x}+fV_{2}$. (4b)

From those equations (4) one can observe that the two layers are coupled by the sloping effect of the upper layer (i.e., $\frac{\partial\eta}{\partial x}\neq0$). The exchange of momentum between the sea surface and the oil slick can indeed be represented by coupling (4a) and (4b). This leads to

$\frac{\partial}{\partial t}\left(U_{2}-U_{1}\right)+\frac{\partial}{\partial x}\left[\frac{1}{2}\left(U_{2}^{2}-U_{1}^{2}\right)+g'h\right]=F_{2}^{x}-F_{1}^{x}+f\left(V_{2}-V_{1}\right)$, (5)

where $g'=g\left(\rho_{2}-\rho_{1}\right)/\rho$ is the reduced gravity. Equation (5) describes the relative seaward (i.e., non-geostrophic) motion between the sea water surface and the oil slick as resulting from SSL spatial variations, frictional effects that may include mixing and wind stress, and the alongshore geostrophic motion of the water layer. Finally, let us note that the structure of equation (5) suggests that we consider the geostrophic alongshore component of the water and oil velocity (i.e., *V*_2_ and *V*_1_) as an additional external force, a sort of known parameter that can be taken from a geostrophic field predicted by numerical simulations. However, if we reasonably assume that both sea water and oil slick have the same alongshore velocity, as given by the geostrophic balance, it results that the Coriolis term in (5) is equal to zero.

A classical parametrization for the frictional terms$F_{i}^{x}$ [17,18] gives:

$F_{1}=E(U_{2}-U_{1})^{2}$ (6)

and

$F_{2}=KU_{2}^{2}$, (7)

where *E* is a stress parameter related to the drag processes occurring between the sea surface water and the oil slick [12,14], and *K* is a bottom drag coefficient [14,19], associated with the bed friction of the water layer. As pointed out by *Astraldi et al.* [14], the determination of these coefficients may be a rather difficult task, especially considering in this case that the chemical/mechanical interactions between the oil slick and the water for the DH spill are as yet unknown. A scale analysis of equation (5) assumes the following values: *O*(*U_i_, V_i_*)~ 10^-2^ m/s (from slick images), *g*'~10^-1^ m/s^2^ (evaluated from a reasonable oil slick density, *ρ*_1_= 600 kg/m^3^), *L*~ 10^3^– 10^4^ m (from slick images), *f* ~ 10^-4^ s^-1^ and *O*(*h*) ~ 10^-2^ m (Figure 2). This suggests a drag coefficient *E* ~ 10^-3^ m^-1^.

In order to better identify the role of river tilting of the SSL in detaching the oil slick from the shore, which corresponds to a positive offshore velocity *U*_1_, one can assume *U*_2_ = 0 and *V*_2_ = 0. In physical terms, this would mean that we are seeking an additional seaward motion that must be superimposed on the main circulation of the Gulf of Mexico. In such a case, one obtains

$\frac{\partial}{\partial t}U_{1}+\frac{\partial}{\partial x}\left[\frac{1}{2}U_{1}^{2}+g'h\right]=-EU_{1}^{2}$. (8)

By choosing a linear *h* = *h*(*x*) that schematically may represent the SSL seaward profile (Figure 5B), equation (8) can be solved for *U*_1_(*x*, *t*) in order to obtain the seaward spatial and temporal evolution of the oil slick (Figure 5C).

We assume a velocity close to zero as initial and boundary conditions, and a sea surface slope ~10^-6^, in accordance with the performed scale analysis, modeled temperature data (Figure S4) and the observed ADT (Figure 2, Figure 3). All this gives an oil slick velocity increasing seaward through time (Figure 5C). One important feature to note is the ability of the system to reach a steady state after a certain amount of time, roughly 5-10 days. A sensitivity analysis made on the unknown drag parameter *E* gives uncertainties of ~30% for variations of Δ*E*/*E* = 50%.

**References**

1. Ocean Observing and Modeling Group (OMG - Department of Marine, Earth & Atmospheric Sciences, North Carolina State University) website. Available: http://omglnx6.meas.ncsu.edu/sabgom_nfcast/. Accessed 2011 April 25.
2. West Florida Shelf Model (WFS ROMS) website. Available: <http://ocgmod1.marine.usf.edu/WFS/>. Accessed 2012 March 30.
3. Naval Research Laboratory website. Available: http://www7320.nrlssc.navy.mil/IASNFS_WWW/IASNFS.html. Accessed 2012 March 30.
4. Mercier F, Rosmorduc, V, Carrere, L., Thibaut P (2010) Coastal and Hydrology Altimetry product (PISTACH) handbook, CLS-DOS-NT-10-246, SALP-MU-P-OP-16031-CN, 01/00, Version 1.0, October 4^th^.
5. Dumont JP, Rosmorduc V, Picot N, Desai S, Bonekamp H, Figa J, Lillibridge J, Scharroo, J (2009) OSTM/Jason-2 Products Handbook, SALP-MU-M-OP-15815-CN, Ed 1.4 .
6. Fu LL, Cazenave A (2001) Satellite altimetry and Earth sciences, A Handbook of techniques and applications. International Geophysics Series 69. Academic, San Diego.
7. Gommenginger C, Thibaut P, Fenoglio-Marc L, Quartly G, Deng X, et al. (2010) Retracking altimeter waveforms near the coasts; A review of retracking methods and some applications to coastal waveforms”, doi: 10.1007/978-3-642-12796-0_6, Springer-Verlag Berlin Heidelberg.
8. Vignudelli S, Kostianoy AG, Cipollini P, Benveniste J (Eds.) (2011) Coastal Altimetry, Springer-Verlag Berlin Heidelberg, doi:10.1007/978-3-642-12796-0, 578 pp.
9. Obligis E, Desportes C, Eymard L, Fernandes J, Lazaro C, Nunes A (2010) Tropospheric corrections for coastal altimetry. In Vignudelli S, Kostianoy AG, Cipollini P, Benveniste J, editors. Coastal Altimetry*.* Berlin Heidelberg, Springer-Verlag, pp. 147−176.
10. Rio MH, Hernandez F (2004) A mean dynamic topography computed over the world ocean from altimetry, in situ measurements, and a geoid model. *J Geophys Res* 109**:** C12032, doi:10.1029/2003JC002226.
11. Bouffard J, Pascual A, Ruiz S, Faugère Y, Tintoré J (2010) Coastal and mesoscale dynamics characterization using altimetry and gliders: A case study in the Balearic Sea. *J Geophys Res* 115: C10029, doi:10.1029/2009JC006087.
12. Pozrikidis C (2001) Fluid Dynamics: Theory, Computation and Numerical Simulation. First Edition (2001), Kluwer.
13. Lane-Serff G, Smeed D, Postlethwaite C (2000) Multi-layer hydraulic exchange flows. *J Fluid Mech* 416: 269-296.
14. Astraldi M, Gasparini G, Gervasio L, Salusti E (2001) Dense water dynamics along the Strait of Sicily (Mediterranean Sea). *J Phys Ocean* 31: 3457-3475.
15. Karadogan E, Willson CS, Berger CR (2009) Numerical modeling of the Lower Mississippi River-influence of forcings on flow distribution and impact of sea level rise on the system, *OCEANS 2009, MTS/IEEE Biloxi - Marine Technology for Our Future: Global and Local Challenges*: 1-7.
16. Gill A. (1977) Hydraulics of rotating-channel flow. *J Fluid Mech* 80: 641-671.
17. Baringer M, Price J (1997a) Mixing and spreading of the Mediterranean outflow *J. Phys Ocean* 27: 1654-1677.
18. Baringer M, Price J (1997b) Momentum and energy balance of the Mediterranean outflow. *J Phys Ocean* 27: 1678-1692.
19. Smith PC (1975) A streamtube model for bottom boundary currents in the ocean, *Deep Sea Res Ocean Abstracts* 22: 853-873.

20. Ko DS, Preller RH, Martin PJ (2003) An Experimental Real-Time Intra Americas Sea Ocean Nowcast/Forecast System for Coastal Prediction, Proceedings, AMS 5th Conference on Coastal Atmospheric & Oceanic Prediction & Processes: 97-100.

**Figure Captions**

**Figure S1 NOAA/AVHRR SST data related to Mississippi River high-discharge event.** (A) 1 June and (B) 6 June, 2010 (data processed by the Earth Scan Lab - Coastal Studies Institute, Louisiana State University). Panels have different ranges in scale. Note cool colors corresponding to fresh MR water surrounding the Birdsfoot.

**Figure S2** **NRM IASNFS model nowcast [3] for 0000 UTC 27 May, 1 June, and 6 June 2010 (A, B, and C, respectively) of SSL and (D, E, and F, respectively) SSS.** IASNFS consists an 1/24 degree , 41-level sigma-z data-assimilating ocean model based on NCOM. The model assimilates the synthetic temperature/salinity profiles generated by a data analysis model called MODAS to produce nowcast. Real-time data come from satellite altimeter (Jason-1, ERS-2) SLA and AVHRR SST. Three hourly surface heat fluxes, including solar radiation, wind stresses and sea level air pressure from NOGAPS/FNMOC are applied for surface forcing. The open boundary conditions including SSL, transport, temperature, salinity and currents are provided by the NRL 1/8 degree Global NCOM which is operated daily [20]. Note in panels A, B, C the disagreement between modeled SLA and measured (Figure 2) ADT in the vicinity of the Birdsfoot (the outermost part of the Mississippi Delta, NW of the Deepwater Horizon location).

**Figure S3 Oil slick extent (left) and ADT data (right) for the time period following Hurricane Alex's passage, 2-11 July 2010.** Approximate location of the oil slick provided by ESRI (http://www.esri.com/services/disaster-response), derived from satellite data published by NOAA National Ocean Service – Office of Response and Restoration. Blue colors correspond qualitatively to slick intensity, while red *x* symbols show locations of oil slick landfall on a given day. Surface slick location and concentration are difficult to quantify, so these maps are meant for illustrative purposes only. NAM12 winds are also shown in inset of right column. Note stronger onshore winds push oil slick “uphill” against the SSH gradient in early July, but as winds subside the slick recedes out to sea along SSH gradients.

**Figure S4 Cross-shelf vertical section of sea temperature from the WFSROMS model.** (A) for a low MR discharge period (1 May 2010) and (B) a high MR discharge event (6 June 2010). Geographical position of the vertical section is shown in the boxes. (C) Schematic representation of the MR water surface slope (solid line), and its elevation (*H*) and seaward extension (*L*) during floods (dashed line).
